# Targeting HOX-PBX interactions causes death in oral potentially malignant and squamous carcinoma cells but not normal oral keratinocytes

**DOI:** 10.1186/s12885-018-4622-0

**Published:** 2018-07-06

**Authors:** Christopher Platais, Raghu Radhakrishnan, Sven Niklander Ebensberger, Richard Morgan, Daniel W. Lambert, Keith D. Hunter

**Affiliations:** 1 Integrated Biosciences, School of Clinical Dentistry, University of Sheffield, Sheffield, UK; 2 Facultad de Odontologia, Universidad Andres Bello, av. Valparaiso, 1560 Viña del Mar, Chile; 3 Institute of Cancer Therapeutics, University of Bradford, Bradford, UK; 4 Department of Oral Biology and Pathology, University of Pretoria, Pretoria, South Africa

**Keywords:** HOX genes, Oral Cancer, OSCC, PBX, HXR9, Apoptosis

## Abstract

**Background:**

High HOX gene expression has been described in many cancers, including oral squamous cell carcinoma and the functional roles of these genes are gradually being understood. The pattern of overexpression suggests that inhibition may be useful therapeutically. Inhibition of HOX protein binding to PBX cofactors by the use of synthetic peptides, such as HXR9, results in apoptosis in multiple cancers.

**Methods:**

Activity of the HOX-PBX inhibiting peptide HXR9 was tested in immortalised normal oral (NOK), potentially-malignant (PMOL) and squamous cell carcinoma (OSCC) cells, compared to the inactive peptide CXR9. Cytotoxicity was assessed by LDH assay. Expression of PBX1/2 and c-Fos was assessed by qPCR and western blotting. Apoptosis was assessed by Annexin-V assay.

**Results:**

PMOL and OSCC cells expressed PBX1/2. HOX-PBX inhibition by HXR9 caused death of PMOL and OSCC cells, but not NOKs. HXR9 treatment resulted in apoptosis and increased expression of c-Fos in some cells, whereas CXR9 did not. A correlation was observed between HOX expression and resistance to HXR9.

**Conclusion:**

Inhibition of HOX-PBX interactions causes selective apoptosis of OSCC/PMOL, indicating selective toxicity that may be useful clinically.

## Background

The HOX proteins are an important family of transcription factors that control a wide array of functions in embryogenesis and the maintenance of normal tissue [1, 2]. Their dysregulation has been implicated in the development of a variety of cancers [3–5], including oral squamous cell carcinoma (OSCC) and we have shown that these changes also occur in cells from potentially malignant oral lesions (PMOLs) [6, 7]. Recent studies have highlighted the therapeutic potential of inhibiting the interaction between HOX proteins and a common group of co-factors, the PBX proteins, using the synthetic peptide HXR9 [8]. The consistent phenotypic effect reported on inhibition of HOX-PBX interactions is induction of apoptosis that has been reported in a number of malignant cell types [9–13]. This has been associated with upregulation of c-Fos protein, which, whilst most often regarded as a proto-oncogene, may in some circumstances be pro-apoptotic [14].

This project aimed to determine whether targeting HOX-PBX interactions has therapeutic potential in OSCCs and PMOLs by assessing the expression of PBX1 and PBX2 in PMOL and OSCC cells and investigating the effect of HOX-PBX inhibition on these cells and in normal oral keratinocytes.

## Methods

### Cell culture

Two immortalised normal oral keratinocyte (iNOK: FNB6TERT and OKF4), two PMOL (D19 and D35) and four OSCC (B16, B22, B56, T5) cell lines were used in this project, as outlined in Table 1. Cell lines FNB6TERT, D19, D35, B16, B22, B56 and T5 were supplied from the Beatson Institute of Cancer Research cell culture collection and OKF4 was supplied by Dr. J Rheinwald (Harvard University, Boston). These cells have been previously extensively characterised [15] and were cultured to a maximum of 70% confluence in keratinocyte growth medium (KGM). Two PMOL (D19, D35) and two OSCC (B16, B22) cell lines were used for the peptide treatment experiments.

**Table 1:** Clinical details relating to the PMOL and OSCC cell lines used in the project. All patients (except B22, unknown status), were smokers. The primary site for B22 was the lateral tongue

Cell line	Age/Gender	Site	Histology	Stage (pTNM)
B16	48 M	Lateral tongue	SCC	T2 N0 M0
B22	88 M	Lymph node metastasis	SCC	T4 N3 M0
B56	59 F	Lateral tongue	SCC	T4 N1 M0
T5	59 F	Buccal mucosa	SCC	T2 N2 M0
D19	53 M	Lateral tongue leukoplakia	Severe dysplasia	N/A
D35	68 M	Lateral tongue erythro-leukoplakia	Severe dysplasia	N/A

*SCC* Squamous cell carcinoma

### RNA isolation and qRT-PCR

Expression of c-Fos and the HOX cofactors PBX1 and PBX2 was assessed using RNA extracted from cells with the Isolate II RNA Mini Kit (Bioline, UK), following the manufacturer’s instructions. Following cDNA generation, the transcript levels of PBX1 and PBX2 were measured using SYBR Green qPCR (Primer sequences: PBX1 forward: 5’ ATTGCAATCCCCCTGCCTTC 3′ reverse: 5’ TTCAGTCCGGTCTCCTTTGC 3′; PBX2 forward: 5’ GATGTACAGCCCACGGGAAA 3′ reverse: 5’ CCGTTGGGGATGTCACTGAA 3′) on a 7900HT Fast Real-Time PCR System (Life Technologies, UK). The expression of c-Fos was assessed using SYBR Green qPCR (Primer sequences - forward: 5’ CCAACCTGCTGAAGGAGAAG 3′ and reverse: 5’ GCTGCTGATGCTCTTGACAG 3′). Data is presented relative to expression of U6. Published expression data for all 39 HOX genes was used to assess possible relationships between peptide sensitivity and HOX gene expression [6].

### Peptide treatment

The HOX-PBX interfering peptide HXR9 and control peptide (CXR9) were custom synthesised by Bio-Synthesis Inc., (Lewisville, Tx, USA), D-isomer to > 90% purity. HXR9: WYPWMKKHHRRRRRRRRR (2700.06 Da), CXR9: WYPAMKKHHRRRRRRRRR (differs from HXR9 by a single amino acid [16], 2604.14 Da). The EC50 of HXR9 and CXR9 was calculated for FNB6TERT, OKF4, D19, D35, B16 and B22 cells using increasing doses of peptide (0.5, 5, 12.5, 25, 50, 75 and 100 μM).

### LDH assay

Cell death was assessed using a lactate dehydrogenase (LDH) cytotoxicity assay (Promega, UK) after 2 h 45 min of peptide treatment, according to the manufacturer’s instructions.

### Annexin–V assay

The induction of apoptosis at EC50 was investigated using the Annexin-V FITC flow cytometry assay (Trevigen, UK) according to the manufacturer’s instructions, using a LSR II flow cytometer (BD Biosciences, San Jose, CA, USA). Gating was applied to the scatter plots to identify cells as viable, early apoptotic, late apoptotic or necrotic. The position of the gate and the quadrants were kept constant between plots of the same cell type, so that the proportions could be compared between treatments.

### Western blot

Western blotting of whole cell lysate (generated using RIPA buffer) was used to assess expression of PBX1 and PBX2 protein. The antibodies used were anti-PBX1: Abcam ab154285 at 1:500, anti-PBX2: Abcam ab55498 at 1:500, and anti-c-Fos (Abcam; ab209794 at 1:100). HeLa whole cell lysate was used as a positive control.

### Statistical methods

Statistical analysis was conducted using ANOVA to assess differences between the expression of these markers in the cell lines tested. The correlation between HOX gene expression and PBX expression was assessed by calculating the Spearman Correlation coefficient. Differences were considered significant if *p* < 0.05.

## Results

### PMOL and OSCC cells express PBX1 and PBX2

**Fig. 1:**
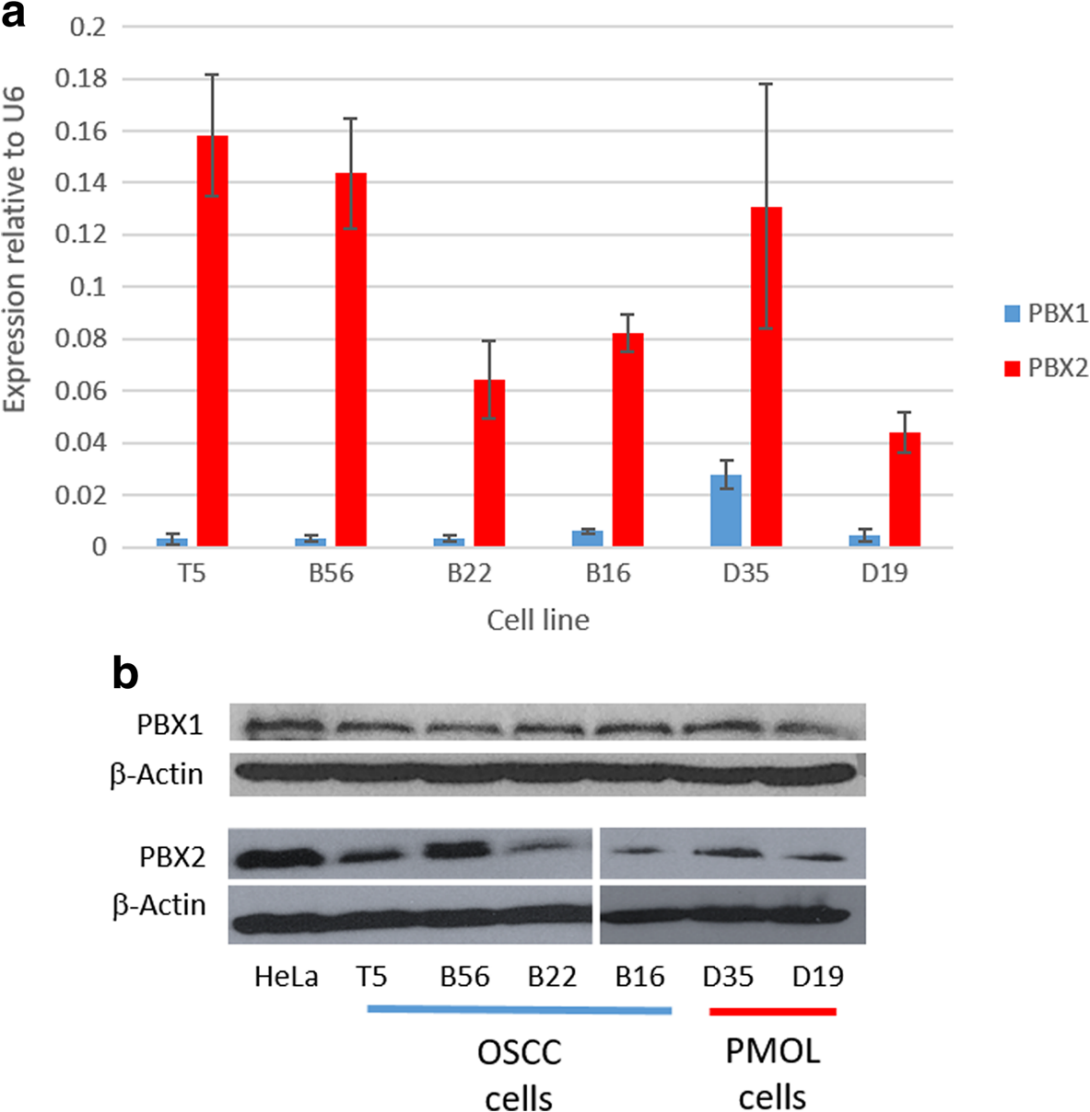
Expression of PBX1 and PBX2 in a panel of PMOL (D19 and D35) and OSCC (B16, B22, B56 and T5) cell lines. Panel **a**: qPCR data, mean±SEM from three individual experiments. Panel **b**: western blot, with β-actin as a loading control

Investigation of PBX1 and PBX2 mRNA and protein expression in PMOL and OSSC cells demonstrated that all cell lines express both PBX1 and PBX2 (Fig. 1a and b). The expression is variable and the relative expression of PBX2 is higher than PBX1. Expression of PBX1 is significantly higher in D35 than in the other cell lines (*p* < 0.05). The expression of PBX proteins largely corresponds to that of the mRNA expression.

### Treatment with HXR9, but not CXR9, results in death of PMOL and OSCC cells

**Fig. 2:**
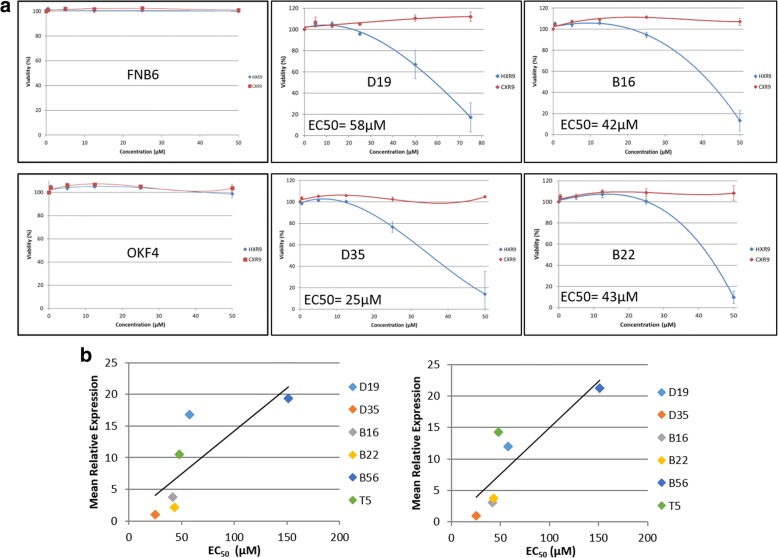
Panel **a**: Relative viability of PMOL, OSCC cells and iNOKs (FNB6 and OKF4) after treatment with increasing doses of HXR9 and control peptide (CXR9; Mean±SEM from three independent experiments). The calculated EC50 stated for each cell line. Panel **b**: Correlation of expression of EC50 with mean expression of genes from all four HOX clusters (left panel; Spearman Coefficient 0.943, *p* < 0.01) and HOXA cluster genes (right panel: Spearman Coefficient 0.943, *p* < 0.01)

HXR9 causes dose dependent death of both PMOL and OSCC cells, whilst iNOKs are insensitive in the concentration range used (Fig. 2a). The control peptide CXR9 has no effect on cell viability in the same concentration range. The EC50 of T5 and B56 is 48 μM and 151 μM SCC: squamous cell carcinomarespectively (data not shown). The sensitivity of the OSCC and PMOL cells varies, with D35 most sensitive and B56 relatively insensitive. Correlation of EC50 values with mRNA expression of PBX1, PBX2 or expression of HOXA, HOXB, HOXC and HOXD demonstrated that the EC50 was positively correlated with the mean expression of all HOX 39 genes and gene expression of each individual HOX cluster (Fig. 2b). There was no relationship between PBX1 or PBX2 expression and EC50.

### HXR9 induces apoptosis and increased expression of cFOS

**Fig. 3:**
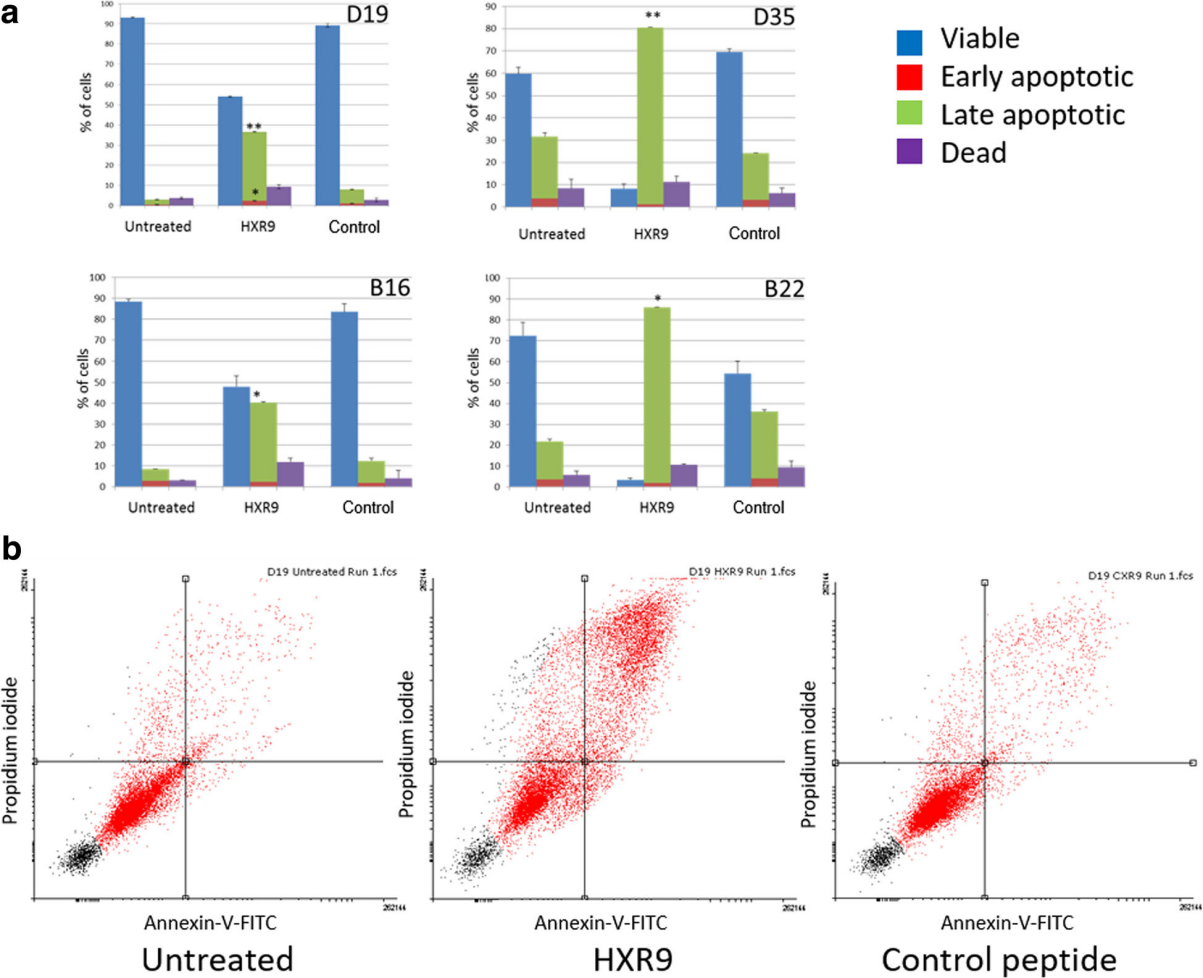
Panel **a**: Induction of apoptosis (assessed by translocation of phosphatidylserine by Annexin-V) in untreated cells and on treatment of cells with CXR9 and HXR9 at EC50 for 2 h 45 min. Blue = viable, red = early apoptotic, green = late apoptotic and purple = dead. Comparisons are of % of late apoptotic cells: Mean±SEM from three individual experiments. *p < 0.05, **p < 0.01. Panel **b**: Exemplar scatter plots for the PMOL cell line D19 cells: untreated, HXR9 treated and control (CXR9) treated. Each quadrant represents a cell status; clockwise from upper left: dead, late apoptotic, early apoptotic and viable

Despite variations in the proportion of apoptosis cells in the untreated cultures, assessment of induction of apoptosis demonstrated that treatment with HXR9 resulted in a significantly higher proportion of cells in late apoptosis when compared to those treated with CXR9 (Fig. 3a). Expression of *c-Fos* mRNA increased after treatment with HXR9 in all cells to a far greater extent than in CXR9 treated cells (Fig. 3b). However, expression of c-Fos protein only increased in B16 and D19 cells, albeit these cells also showed the largest increase in mRNA expression (Fig. 3c).

## Discussion

Identification of effective molecularly based therapeutics is vital if similar breakthroughs are to be made in the treatment of OSCC as in other solid tumours such as breast cancer. The observation that dysregulation of HOX gene expression occurs early in the pathogenesis of OSCC makes this family of transcription factors an attractive therapeutic target [7]. The main challenge in targeting HOX genes is their functional redundancy and the relatively conserved DNA binding site, thus specifically targeting individual HOX genes is unlikely to be feasible. The discovery that many HOX genes (particularly paralogous groups 1–8) require co-factor binding for stable interaction with DNA opens the potential for interfering with this interaction pharmaceutically using the HXR9 peptide. The effects of HXR9 have been demonstrated in a number of solid malignancies in vitro and in vivo [9, 11, 12, 17]. In this paper, we demonstrate the sensitivity of oral cancer cells to this agent, similar to that reported for other cancers, but in addition, we demonstrate similar effects on cells derived from potentially malignant oral lesions and the lack of effect on normal oral keratinocytes. This measure of selectivity was not seen on comparison of breast cancer cell lines with MCF10A cells where the sensitivity of three of the five cell lines tested was no different from the non-malignant MCF10A cells [11].

The data also suggests that HOX-PBX inhibition may be an effective strategy to prevent the development of OSCC due to its selective toxicity in PMOL cells. This may have particular benefit in the treatment of large PMOLs, which are not amenable to surgery, and for the prevention of subsequent malignancies in patients who have already developed an upper aero-digestive tract malignancy. These are significant clinical issues in the management of patients with PMOLs and OSCC.

The sensitivity of PMOL and OSCC cells is correlated with HOX gene expression overall and the median expression of the individual clusters, most particularly HOXA cluster genes (paralogues 1–8). Despite modest variation in expression of PBX1 and PBX2, there is no relationship between this and the EC50 of HXR9 in these cells. Presumably, this indicates that PBX co-factors are present in excess and as such, it is the aberrant expression of HOX genes in OSCC pathogenesis that confers the specificity. There was no effect on NOKs at the concentrations tested.

The mechanism of cell death induced by HXR9 has been demonstrated to be apoptosis in other solid malignancies [9, 11, 12, 17]. Similarly, we found that treatment of PMOL and OSCC cells with HXR9 resulted in induction of apoptosis with a significant increase in the number of cells in late apoptosis (Fig. 3).

**Fig. 4:**
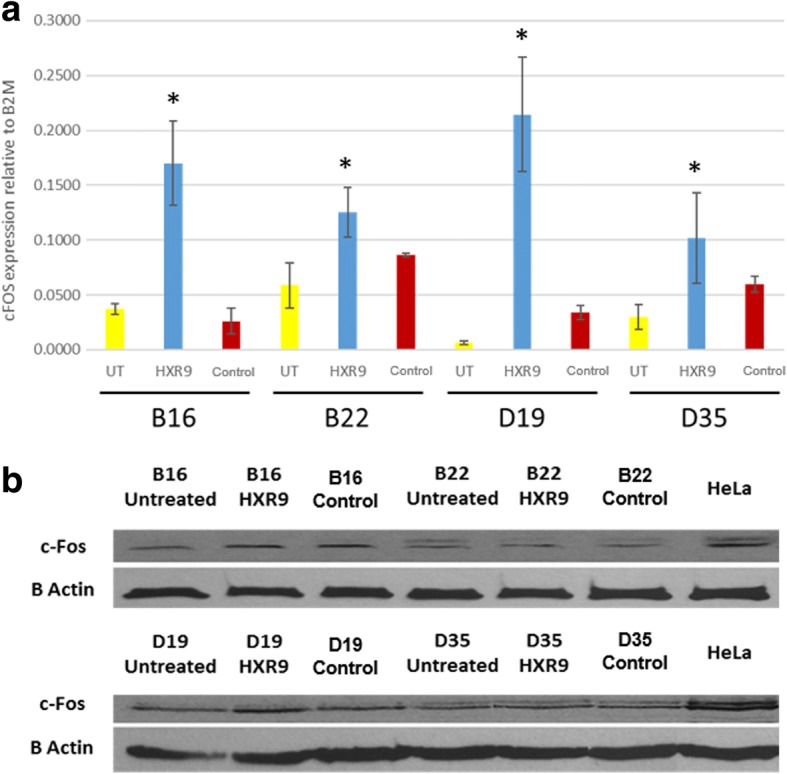
Panel **a**: Expression of c-Fos by qPCR untreated cells and cells treated with HXR9 or control peptide (CXR9) at EC50. Mean±SEM from three independent experiments, relative to U6. *p < 0.05. Panel **b**: Expression of c-Fos by western blot in untreated cells and cells treated with HXR9 or control peptide (CXR9) at EC50. β-actin was used as a loading control

HXR9 also increased c-Fos expression, which has been reported in other tumours, such as melanoma and prostate cancer [8, 10] (Fig. 4). Whilst mRNA expression increased markedly after HXR9 treatment, the increase in the expression of c-Fos protein is less consistent. Both the mRNA transcript and protein products are short lived and undergo rapid degradation. This indicates that further studies are required on the dynamics of c-Fos expression in these cells after treatment with HXR9.

## Conclusion

These data demonstrate the therapeutic potential of HOX-PBX inhibitors not only in OSCC, but also in PMOLs, suggesting a wide range of possible therapeutic uses. This is a specific effect on cells with dysregulated HOX gene expression and iNOKs are not affected.
